# Biocompatibility and bioactivity of bioceramic sealers containing 1% cetrimide

**DOI:** 10.1590/1807-3107bor-2025.vol39.042

**Published:** 2025-05-12

**Authors:** Evelin Carine Alves SILVA, Jéssica Arielli PRADELLI, Guilherme Ferreira da SILVA, Paulo Sérgio CERRI, Mario TANOMARU-FILHO, Juliane Maria GUERREIRO-TANOMARU

**Affiliations:** (a) School of Dentistry, Department of Restorative Dentistry, Araraquara, SP, Brazil.; (b) Unisagrado, Department of Dentistry, Bauru, SP, Brazil; (c) Universidade Estadual Paulista – Unesp, School of Dentistry, Department of Morphology, Araraquara, SP, Brazil.

**Keywords:** Biocompatible Materials, Root Canal Filling Materials, Rats, Osteocalcin

## Introduction

The antimicrobial activity has been desirable for repair cements and root canal filling materials and may collaborate for the control of residual infection.^1-3^ Thus, the incorporation of antimicrobial agents into endodontic materials can improve their effect against remaining microorganisms. Cetrimide (CTR) is a cationic surfactant, which has demonstrated efficacy against gram-positive and gram-negative bacteria.^2-4^ CTR was incorporated into an experimental bioceramic cement formulated with pure tricalciumsilicate and zirconium oxide as a radiopacifier (TSC/ZrO2). The highest antibiofilm activity was observed in the TSC/ZrO2+CTR, and the physicochemical properties such as setting time, pH, and solubility were maintained, according to the ISO-6876 standard.^3^

However, in vivo studies are needed to evaluate whether CTR impairs the tissue response when added to the endodontic sealers. Endodontic sealers should ideally present biocompatibility and bioactive potential to stimulate periapical repair.^4,5^ BioRoot™ RCS (BROOT; Septodont, Saint-Maur-des-Fossés, France) is a calcium silicate-based bioceramic sealer in powder/liquid composition. The biocompatibility of BioRoot RCS has been reported in several in vitro studies, where the effects on cells depend on the dilution of their extracts and time of exposure.^4,5^ Chlorhexidine improved the antibacterial activity of bioceramic sealers such as BioRoot, but decreased cell viability, in addition to affecting their physicochemical properties.^6^ Therefore, the association of bioceramic sealers with antimicrobial agents with the aim of achieving better antimicrobial activity can be beneficial, as it does not alter physicochemical and biological properties.

NeoSealer Flo (NeoFlo, NuSmile, Houston, TX, USA) is a new ready-to-use bioceramic endodontic sealer composed of dicalcium and tricalcium silicate. NeoSealer Flo showed radiopacity, proper setting time, film thickness and alkaline pH up to 28 days, in addition to exhibiting the formation of a thin layer of phosphate phase calcium.^7^

The aim of this study was to assess the biocompatibility and bioactive potential of the commercial bioceramic materials BioRoot™ RCS and NeoSealer Flo, and their associations with cetrimide. The null hypothesis was that the addition of cetrimide would not interfere with the tissue reaction and bioactive potential of the sealers.

## Methods

Ethical approval for the study was obtained (Protocol #19/2021) Holtzman rats (Rattus norvegicus albinus) weighing ± 250-300 g were randomly distributed into 5 groups (n = 6/group), according to the sealers evaluated (Table 1) and a control group (GC; empty polyethylene tubes). The animals were kept in controlled temperature, humidity accommodation, and had access to food and water ad libitum. The authors conducted the study in compliance with the ARRIVE (Animal Research: Reporting of In Vivo Experiments) guidelines .

**Table 1:** Endodontic sealers and experimental materials

Sealers and manufacturers	Proportion	Composition
NeoSealer	Ready to use	Dicalcium and tricalcium silicate with calcium aluminate, tricalcium aluminate, and tantalite and thickening agent.
Flo (NeoFlo)/NuSmile, Houston (USA)		
+ CTR 1% (cetrimide, # (Sigma-Aldrich, USA)	990 mg of sealer NeoSealer and 10 mg CTR	
BioRoot RCS/ Septodont (Saint-Maur-des-Fossé, France).	1 portion of dust and 5 drops of liquid according to the manufacturer, with measurement on a scale	Powder: Knitting silicate, zirconium oxide, and povidone. Liquid: dihydrate calcium chloride, sand, purified water
+ CTR 1% (cetrimide, # (Sigma-Aldrich, USA)	990 mg of already manipulated sealer BioRoot and 10 mg CTR	

The animals were anesthetized with ketamine chloride (80 mg/kg, Virbac do Brasil Indústria e Comércio Ltda., Brazil) and xylazine chloride (8 mg/kg, National Pharmaceutical Chemical Union, Brazil) by intraperitoneal administration. After shaving the dorsal region, the skin was disinfected using 5% iodine solution. Subsequently, a 1 cm incision in the craniocaudal direction was made with a scalpel blade. The tissues were dissected with blunt-tipped scissors to create a pocket in the subcutaneous tissue. Polyethylene tubes, approximately 10 mm in length and 1.6 mm in diameter, were then filled with the respective materials (Table 1). All materials were manipulated in accordance with the manufacturers’ recommendations. BROOT sealer was prepared using a ratio of 1 part powder to 5 drops of liquid, and 10 mg of CTR was added for every 990 mg of material processed. Similarly, manipulation was performed for NeoFlo associated with CTR. In the GC, empty polyethylene tubes were used. Six rats were used in each period for each group, including an additional animal due to the possibility of eventual loss during the experiment. Four tubes were inserted per animal, one from each group, following a quadrant rotation (ISO-10993-6). After 7, 15, 30, and 60 days after implantation, the animals were sacrificed with an overdose dose of ketamine chloride and xylazine chloride. The implants with adjacent tissues were removed and samples were fixed with 4% buffered formaldehyde at pH 7.2.^8,9^

After 48 h, the samples were dehydrated, treated with xylene, immersed in liquid paraffin, and embedded in paraffin to obtain longitudinal sections of the implants surrounded by capsules for analysis of both ends. Serial sections were obtained for hematoxylin & eosin (HE), picrosirius-red, von Kossa reaction staining, and immunohistochemistry for the detection of osteocalcin (OCN).

### Thickness of capsules around implants

In each specimen, the thickness of capsules was estimated from three HE-stained non-serial sections. The thickness of the capsules was measured in the middle portion from its innermost surface adjacent to the material until its limit with adjacent tissues.^8,9^ According to thickness, the capsules were characterized as thin when measured below 150 µm and thick when exhibiting values above 150 µm.^8^

### Numerical density of inflammatory cells and fibroblasts

Quantitative analysis of inflammatory cells (IC) and fibroblasts was conducted in the capsules of all implants. In each specimen, 3 HE-stained non-serial sections of portions of the capsule adjacent to the opening of the polyethylene tubes were captured at x545 magnification, totaling a standard field of 0.27 mm^2^. The number of IC and fibroblasts was computed using an image analysis software (Olympus Image-Pro Express 6.0, Tokyo, Japan). IC (neutrophils, lymphocytes, plasma cells, and macrophages) and fibroblasts were identified based on their morphological characteristics. Inflammatory cells were distinguished by their rounded or ovoid shapes and specific nuclear features, while the fibroblast was recognized as a fusiform cell with an elliptical nucleus.

The inflammatory reaction intensity was estimated according to the number of IC per field,^9,10^ the inflammatory reaction was classified as mild (containing up to 25 IC/field), moderate (containing from 26 to 125 IC /field), and severe/intense (containing more than 125 IC /field).

### The content of collagen in the capsules

In each specimen, the amount of birefringent collagen was analyzed from three non-serial sections stained with 0.1% picrosirius-red solution. Using a polarized light microscope, a field of the capsule was captured (at ×40 objective lens) with rigorously standardized parameters (light intensity, diaphragm aperture, condenser position, and exposure time). The birefringent collagen was computed using image analysis software (ImageJ; National Health Institutes; Bethesda, USA).^11,12^

### Immunohistochemical detection of OCN

Paraffin-free sections were treated with sodium citrate and heated (98ºC, 30 min) in a microwave oven. After washings, sections were exposed to 5% hydrogen peroxide and 2% bovine serum albumin. Subsequent to incubation, the procedure continued was rabbit anti-OCN primary antibody (1:150) at 4ºC. Sections were processed using a Labelled StreptAvidin-Biotin Kit, and peroxidase activity was revealed with 3.3’-diaminobenzidine. For counterstaining Carazzi’s hematoxylin was used Immunolabelled cells/mm2 were quantified via Image-Pro Express 6.0 software. A standardized field was captured (x40 objective lens) using a DP-71 digital camera attached to an Olympus microscope. The blinded examiner computed the number of OCN-immunolabelled cells/mm^2^ in the images.

### Von Kossa reaction and analysis under polarized light

Sections were dewaxed and treated with 5% silver nitrate for 1 hour under incandescent light. After washings, slides were immersed in 5% sodium hyposulfite for 5 min. Sections were then stained with 0.1% picrosirius-red solution and mounted. Additional unstained sections, adjacent to those treated with von Kossa reaction, were dewaxed, dehydrated, and examined under a light microscope with polarization filters (Olympus, BX51).

### Statistical analysis

Data were analyzed using a two-way ANOVA to assess the impact of treatments and time intervals, followed by Tukey’s post-hoc test to identify specific group variations, with significance set at p ≤ 0.05. Osteocalcin data were submitted to the Kruskal-Wallis test for group comparisons, followed by Dunn’s post-hoc test for detailed pairwise comparisons. Friedman’s test was used for repeated measures analysis, and Nemenyi’s post-hoc test was conducted to evaluate differences across different time intervals.

## Results

### Morphological findings, capsule thickness, and numerical density of IC and FB

**Figure 1:**
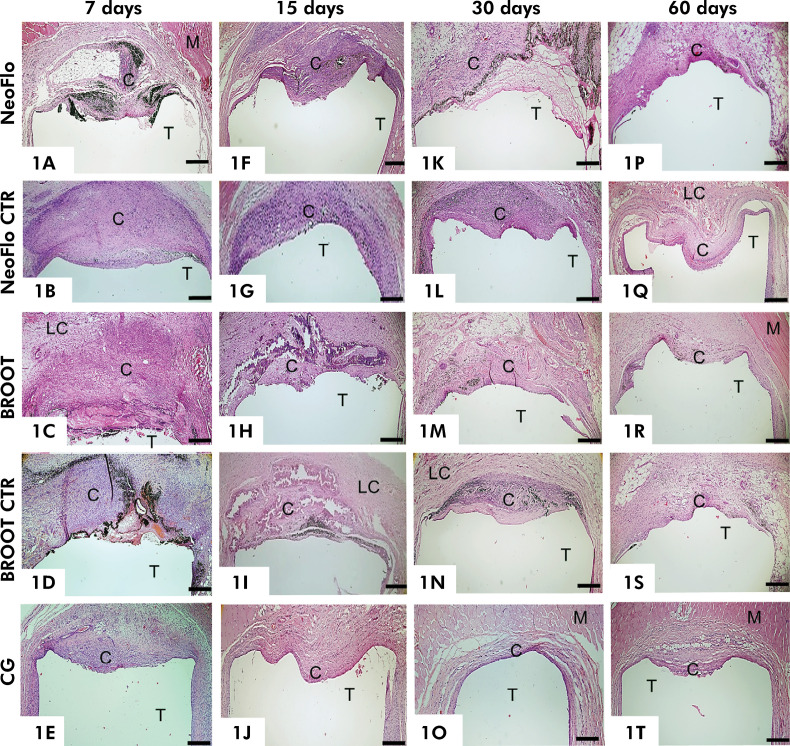
Photomicrographs show a general view of capsules (C) adjacent to the opening of the tubes implanted (T). At 7 and 15 days, thick capsules (C) containing numerous inflammatory cells are seen around the implanted materials (Figs. 1A-1D and 1F-1I) in comparison with CG specimens (Figs. 1E and 1J). After 30 days of implantation (Figs. 1K-1O), a thick capsule with strong basophilia is observed juxtaposed to the NeoFlo CTR material (Fig. 1L). At 60 days, well-defined capsules are seen around all specimens (Figs. 1P-1T). * Demonstrates the central portion used to measure the capsule thickness. M, muscle tissue; LC, loose connective tissue. Scale bars: 900 μm.

After 7 and 15 days of implantation, thick capsules with numerous IC surrounded the implanted materials (Figures 1A-1D and 1F-1I). Even after 30 days (Figures 1K-1N), capsules with a dense IC population were still present, particularly adjacent to NeoFlo CTR material. By 60 days, all specimens exhibited well-defined connective tissue capsules (Figures 1P-1T). At 7 and 15 days, the thickness of capsules around NeoFlo CTR and BROOT CTR specimens was greater than it was in the capsules of NeoFlo and BROOT pure materials (Table 2). At 30 days, no significant difference in the thickness of capsules was observed among NeoFlo, BROOT, and BROOT CTR specimens while the highest values were noted in NeoFlo CTR. By 60 days, no significant differences in capsule thickness were noted between the materials.

**Table 2:** Thickness of capsules (TC), number of inflammatory cells (ICs), number of fibroblasts (Fb), number of OCN-immunolabelled cells, collagen content (CF) and inflammatory reaction intensity (IR) in the capsules around the NeoSealer Flo (NeoFlo), NeoSealer Flo + Cetrimide (NeoFlo CTR), BioRoot (BROOT), BioRoot + Cetrimide (BROOT CTR) and Control group (CG) at 7, 15, 30 e 60 days of implantation.

Periods (days)	Analyses	NeoFlo	NeoFlo CTR	BROOT	BROOT CTR	CG
7	TC (µm)	366 ± 43^a;1^	733 ± 24^b;1^	489 ± 25^c;1^	772 ± 25^b;1^	168 ± 13^d;1^
	ICs/mm^2^	1569 ± 27^a;1^	1650 ± 15^b;1^	971 ± 19 ^c;1^	1555 ± 22^a;1^	249 ± 32^d;1^
	Fb/mm^2^	104 ± 21 ^a;1^	93 ± 16 ^b;1^	129 ± 16 ^a;1^	80 ± 9 ^b;1^	168 ± 10 ^c;1^
	CF (%)	13.1 ± 4 ^a;1^	9.7± 1 ^b;1^	14.9 ± 2 ^c;1^	5.9 ± 1 ^c;1^	16.4 ± 2 ^a;1^
	OCN/mm^2^	11.11(11.11) ^a;1^	0.00(0.0) ^b;1^	0.00(0.0) ^b;1^	0.00(0.0) ^b;1^	0.00 (0.0) ^b;1^
	IR	intense	intense	intense	intense	mild
15	TC (µm)	323 ± 40^a;1^	491 ± 26^b;2^	439 ± 20^b;1^	504± 18^c;2^	181 ± 11^d;1^
	ICs/mm^2^	1429 ± 21^a;1^	1324 ± 12^b;2^	902 ± 10^c;1^	1012 ± 26^d;2^	233 ± 14^e;1^
	Fb/mm^2^	151 ± 21^a;2^	114 ± 12 ^b;2^	141 ± 6 ^a;1^	130 ± 9 ^a;2^	236± 21 ^c;2^
	CF (%)	20.6 ± 3 ^a;2^	16.9 ± 1 ^a;2^	18.5 ± 2 ^b;2^	14.8 ± 2 ^b;2^	28.9 ± 3 ^a;2^
	OCN/mm^2^	22.22(22.22) ^a;2^	0.00(0.0) ^b;1^	0.00(0.0) ^b;1^	0.00(0.0) ^b;1^	0.00(0.0) ^b;1^
	IR	intense	intense	intense	intense	mild
30	TC (µm)	279 ± 41^a;2^	304 ± 22^b;3^	246 ± 24^a;2^	276 ± 20^a;3^	111 ± 03^c,1^
	ICs/mm^2^	990 ± 21^a;2^	1103 ± 22 ^b;3^	684 ± 22^c;2^	804 ± 17^d;3^	175 ± 26^e,2^
	Fb/mm^2^	231 ± 09 ^a;3^	190 ± 9 ^b;3^	196 ± 10 ^b;2^	180 ± 13 ^b;3^	313 ± 14 ^c;3^
	CF (%)	25.8 ± 1 ^a;3^	19.3 ± 1 ^a;3^	20.9 ± 3 ^a;3^	16.6 ± 1 ^b;2^	26.8 ± 2 ^a;2^
	OCN/mm^2^	22.22(22.22) ^a;2^	0.00(0.0) ^b;1^	0.00(0.0) ^b;1^	0.00(0.0) ^b;1^	0.00(0.0) ^b;1^
	IR	moderate	moderate	moderate	moderate	mild
60	TC (µm)	215 ± 45^a;2^	237 ± 24^a;4^	223 ± 23^a;2^	247 ± 13^a;3^	101 ± 10^b,1^
	ICs/mm^2^	503 ± 24^a;3^	611 ± 15^b;4^	297 ± 23^c;3^	401 ± 15^d;4^	67 ± 11^e,3^
	Fb/mm^2^	360 ± 23 ^a;4^	243 ± 10 ^b;4^	268 ± 16 ^b;3^	232 ± 6 ^b;4^	341 ± 16 ^a;3^
	CF (%)	29.9 ± 2 ^a;3^	26.8 ± 1 ^a;2^	23.2 ± 1 ^b;3^	20.1 ± 4 ^b;3^	29.4 ± 1 ^a;2^
	OCN/mm^2^	33.33(22.22) ^a;3^	22.22(22.22) ^b;2^	11.11(11.1) ^c;2^	11.11(11.11) ^c,2^	0.00(0.0) ^d;1^
	IR	moderate	moderate	moderate	moderate	mild

**Figure 2:**
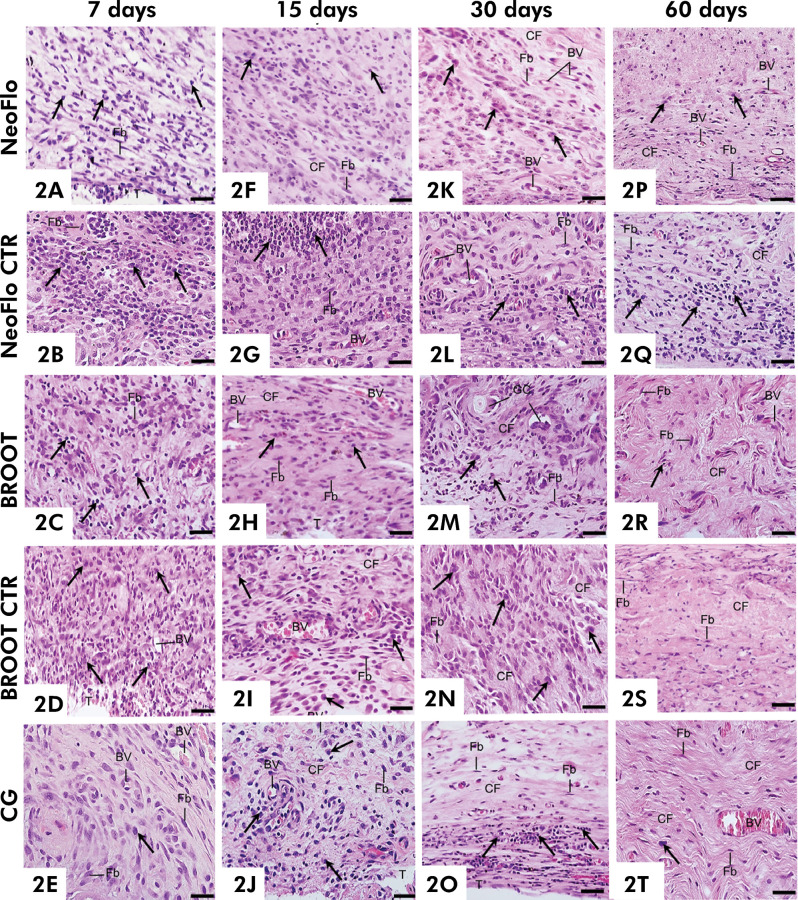
Photomicrographs showing portions of sections of capsules after 7 (2A-2E), 15 (2F-2J), 30 (2K-2O) and 60 days (2P-2T). Several inflammatory cells (arrows) are observed in the capsules around the materials at 7 (2A-2D), 15 (2F-2I) and 30 (2K-2N) days in comparison with CG specimens (2E, 2J and 2O). Giant multinucleated cells (GC) are present in the capsule around BROOT (Fig. 2M). At 60 days, fibroblasts (Fb) among bundles of collagen fibers (CF) and inflammatory cells (arrows) are seen in the capsules. T, space of polyethylene tube. BV, blood vessels. Scale bars: 18 µm.

At high magnification, a massive presence of macrophages and lymphocytes (Figures 2A-2D) was revealed. These capsules exhibited an intense inflammatory reaction, except in the BROOT specimens that presented a moderate inflammatory reaction (Table 2). At 15 days, an intense inflammatory reaction was found in the capsules of NeoFlo and NeoFlo CTR specimens (Figures 2F and 2G) while BROOT and BROOT CTR specimens exhibited moderate reaction (Figures 2H-2I and Table 2). At 30 (Figures 2K-2N) and 60 days (Figures 2P-2S), although the IC were more sparsely distributed in comparison with the periods of 7 (Figures 2A-2D) and 15 (Figures 2F-2I) days, the capsule around all materials still showed a moderate inflammatory reaction (Table 2).

Regarding numerical density (Table 2), the number of IC was significantly greater in NeoFlo CTR than in NeoFlo at 7, 30, and 60 days (p < 0.05) while, at 15 days, the number of IC was significantly lower in the NeoFlo CTR specimens. In all time intervals a greater number of IC was found in the capsules around BROOT CTR than in BROOT specimens. The number of IC was significantly lower in BROOT CTR than in NeoFlo specimens at 15, 30, and 60 days (p < 0.05). In all periods, capsules around BROOT material contained a lower number of IC (p < 0.05) than found in NeoFlo, NeoFlo CTR, and BROOT CTR materials.

After 7 days, the capsules of NeoFlo and BROOT materials had a greater number of fibroblasts than there were in capsules of NeoFlo CTR and BROOT CTR (p < 0.0001); no significant difference was detected between NeoFlo CTR and BROOT CTR. After 15 days, no significant differences in the number of fibroblasts were observed among NeoFlo, BROOT, and BROOT CTR. However, at 30 and 60 days, capsules around the NeoFlo specimens exhibited a higher number of fibroblasts than found in the capsules around other materials.

### The collagen content in the capsules

**Figure 3:**
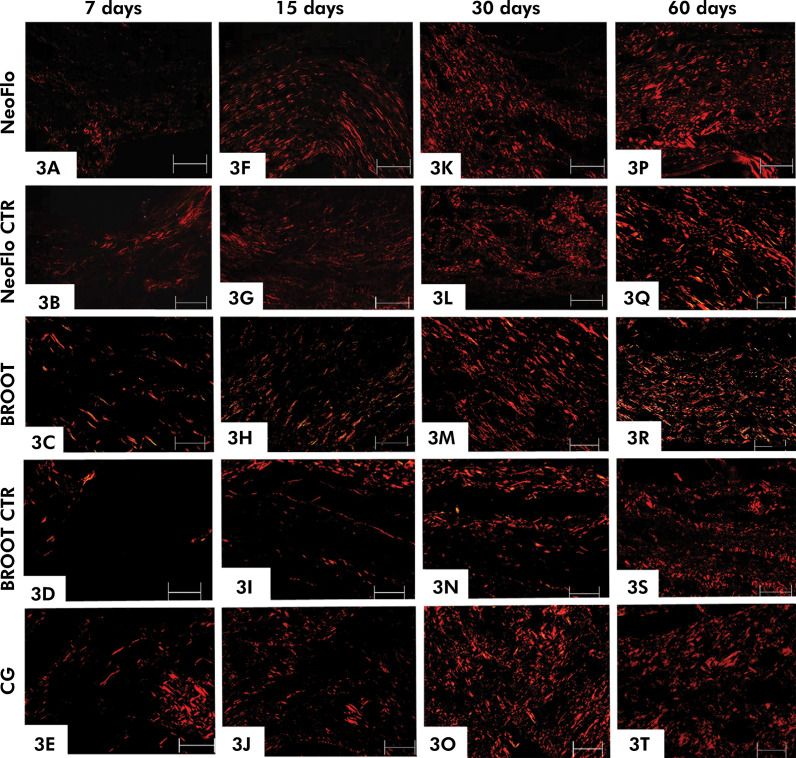
Photomicrographs of sections showing portions of capsules after 7 (3A-3E), 15 (3F-3J), 30 (3K-3O) and 60 (3P-3T) days of implantation. Sections were stained with picrosirius-red and analyzed under polarized light. Birefringent collagen fibers are mainly seen in red color. T, space of polyethylene tube. Scale bars: 20 μm.

As shown in Table 2, a few bundles of birefringent collagen were seen in the capsules of all specimens at 7 days (Figures 3A-3E). However, in all specimens, a significant increase in the amount of birefringent collagen was observed over time (Figures 3A-3P; Table 2).

At 7 days, the collagen content was significantly lower in the BROOT CTR than in BROOT (p < 0.0001), and NeoFlo CTR (p < 0.0001). After 15 days, no significant difference was seen between CG with NeoFlo and with BROOT (p > 0.05), which showed the highest collagen values. In contrast, the lowest values were found in the NeoFlo CTR and BROOT CTR specimens. At 30 days, the percentage of collagen was significantly greater in the NeoFlo, BROOT, and CG specimens than in NeoFlow CTR and BROOT CTR specimens. At 60 days, no significant differences were observed between NeoFlo and NeoFlo CTR, NeoFlo and BROOT, and NeoFlo CTR and BROOT.

### Immunohistochemical detection of OCN

**Figure 4:**
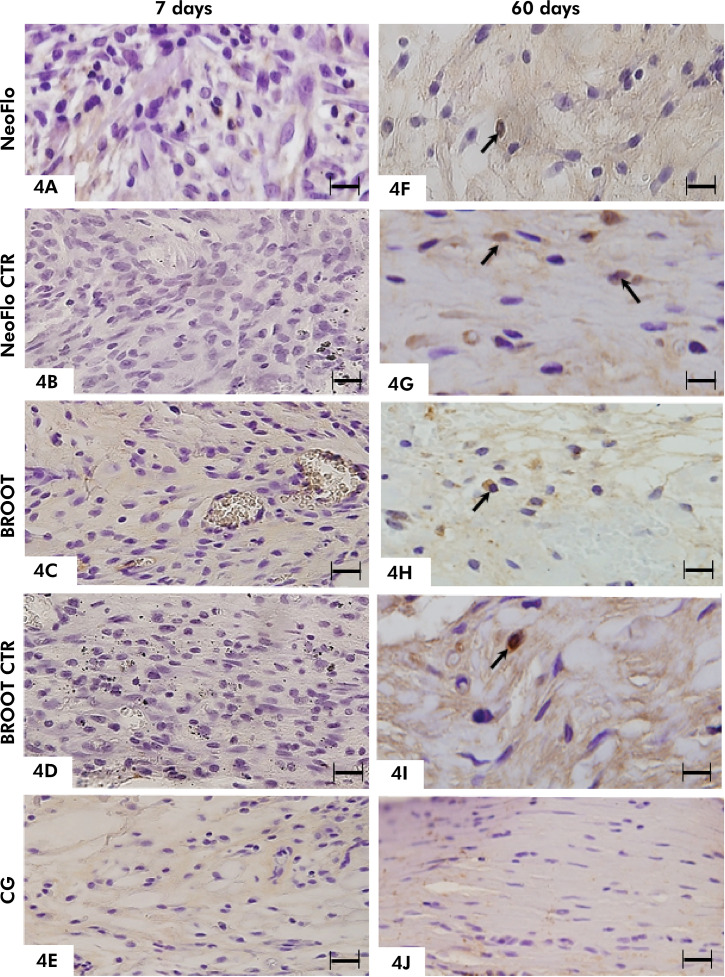
Photomicrographs showing portions of capsules after 7 (4A-4E) and 60 days (4F-4J). Sections were submitted to immunohistochemistry for detection of OCN and counterstained with hematoxylin. At 7 days, OCN-immunostained cell (arrow) is observed in the capsule around NeoFlo sealer (4A) only. At 60 days, immunostained cells (arrows) are seen in the capsules of all sealers (4F-4I). In the capsules around CG specimens (4E and 4J) no immunolabelling is observed. T, space of polyethylene tube. Scale bars: 18 µm.

The immunoexpression of OCN was observed in the capsules surrounding the NeoFlo samples in all time intervals (Figures 4A-4F). Otherwise, OCN-immunolabelled cells in NeoFlo CTR, BROOT, and BROOT CTR specimens were only observed at 60 days (Figures 4F-4I). No OCN-immunolabelled cells were seen in the capsules around CG specimens (Figures 4E-4J; Table 2). As shown in Table 2, at 60 days NeoFlo showed the highest values of OCN-immunolabelled cells, while no significant difference was observed between BROOT and BROOT CTR.

### Von Kossa reaction and analysis under polarized light

**Figure 5:**
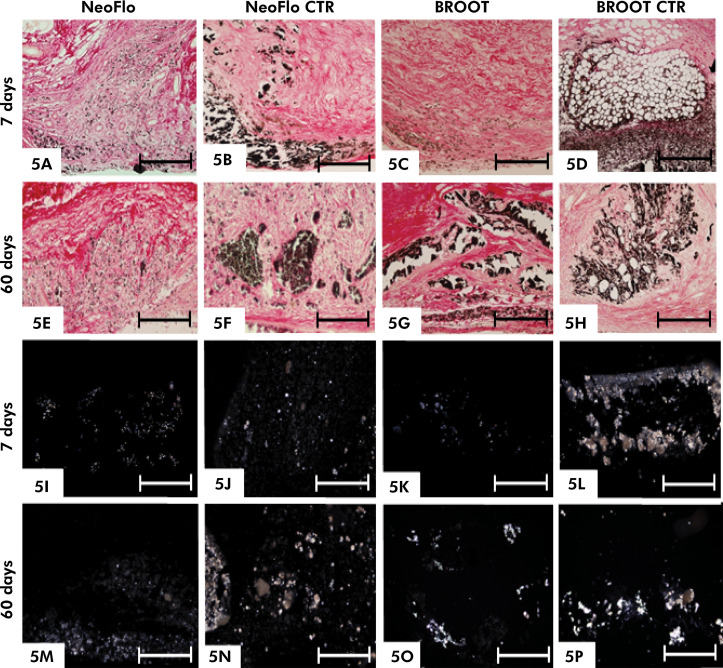
Photomicrographs showing portions of sections of capsule subjected to the von Kossa reaction and counterstained with picrosirius-red after 7 (5A-5D) and 60 (5E-5H) days implantation. von Kossa-positive structures (brown/yellow or black color) are observed in the capsules around the sealers. Small brown/yellow-stained deposits (von Kossa-positive) are dispersed throughout capsules around NeoFlo and BROOT specimens (Figs. 5A, 5C and 5E). In 5B, 5D and 5F-5H irregular structures exhibiting strong positivity for the von Kossa (stained in black) are observed in the capsules. Figs. 5I-5P - Photomicrographs showing portions of unstained sections analyzed under polarized light. Birefringent structures are seen in the capsules. T, space of polyethylene tube. Scale bars: 36 µm.

The capsules around all sealers exhibited von Kossa-positive structures in all time intervals (Figures 5A-5H). Moreover, the analyses of unstained sections performed under polarized light revealed birefringent structures scattered throughout capsules adjacent to the materials (Figures 5I-5P). No von Kossa-positive or birefringent structures were seen in the CG specimens (data not shown).

## Discussion

In the present study the biocompatibility and bioactive potential of BioRoot™ RCS and NeoSealer Flo sealers and their associations with cetrimide (CTR were evaluated). The addition of CTR to bioceramic sealers promoted intense recruitment of IC leading to formation of thick capsules compared with those around pure bioceramic sealers after implantation into the rat subcutaneous connective tissue. Therefore, the null hypothesis was rejected.

The physical and antimicrobial properties of bioceramic sealers, together with the biological properties, are promising and can potentially improve the clinical success of treatment.^13,14^ Bioceramic sealers are used in Endodontics due to their biological properties and bioactivity. The composition of endodontic sealers plays an important role in their biocompatibility and bioactive potential.

Although NeoFlo and BROOT sealers initially induced similar tissue injuries, faster regression of tissue reaction occurred for BROOT compared with NeoFlo, indicating better biocompatibility for BROOT. The higher IC values promoting an intense inflammatory process in the initial period can be explained by alkaline pH and high solubility presented by NeoFlo and BROOT sealers.^4,7^ BROOT presents an alkaline pH until 28 days, and has higher solubility than AH Plus^4^. BioRoot RCS (BROOT; Septodont, Saint-Maur-des-Fossés, France) is composed of a powder that contains tricalcium silicates, zirconium oxide, and povidone, while the liquid contains polycarboxylate and calcium chloride. BROOT releases calcium has alkalizing activity and induces apatite formation.^4^ NeoFlo has calcium sulfate, grossite and tantalite in its composition, which may explain the different tissue reaction patterns between these materials. To date, no studies that evaluate the tissue reaction to NeoSeaaler Flo in vivo have been found.

Endodontic sealers with antibacterial properties can contribute to reduction in residual microbial presence after root canal preparation. However, limited antibacterial activity has been observed for BROOT in direct contact against E. faecalis.^5^ Moreover, BROOT was not able to prevent the formation of different stages of biofilm.^10^ As regards NeoFlo, there are still no studies evaluating its antimicrobial activity.

CTR is a cationic surfactant with antibacterial activity^1-3,15^ that has shown the ability to reduce bacterial adhesion^4^, justifying the choice for its addition to the materials in the present study. Moreover, 1% or 0.5% CTR did not cause significant changes in the setting time, flow, solubility, and radiopacity when added to the AH^15^ Plus. The incorporation of surfactants into endodontic materials has been reported and demonstrated the potential to improve antimicrobial activity.^3^ The concentration of 1% was chosen to provide an antimicrobial effect, without significantly altering the physical and chemical properties of the materials. However, no in vivo study that has evaluated the tissue reaction of CTR added to the bioceramic sealers was found.

In the present study, the addition of 1% CTR to NeoFlo or BROOT promoted greater damage to the connective tissue for the sealers evaluated, especially in the initial periods. The addition of CTR to NeoFlo and BROOT recruited greater numbers of IC, culminating in the maintenance of the inflammatory reaction for a prolonged time. CTR added to V79 Chinese hamster cells caused a high rate of cell death demonstrated by MTT, clonogenic, and micronucleus assays.^16^ It has been suggested that CTR promotes disruption of cell membrane lipids, resulting in cell lysis.^16^

Our findings also revealed that the capsules around all materials exhibited a moderate inflammatory reaction. However, the significant reduction in the number of IC and in the capsule thickness observed over time, suggested that NeoFlo and BROOT sealers as well as the experimental sealers were biocompatible. Although CTR added to the BROOT sealer caused greater tissue injury than pure BROOT, it is important to emphasize that damage promoted by BROOT CTR decreased more quickly in comparison with that promoted by the pure NeoFlo.

Despite the inflammatory reaction, the high values of Ca^2^ and OH^-1^ released by these bioceramic sealers may provide a suitable microenvironment for inducing the differentiation of mesenchymal cells into cells with the typical phenotype of producing mineralized tissue cells.^17^ NeoFlo sealer induced the immunoexpression of OCN by cells of subcutaneous connective tissue at all time intervals. OCN is a small glycoprotein, expressed by differentiating osteoblasts.^17,18^ Studies have shown that calcium silicate-based endodontic materials induce the immunoexpression of OCN suggesting that these materials may stimulate the differentiation of mesenchymal cells into cells able to produce mineralized tissue proteins ^18,19^ Therefore, it is conceivable to suggest that CTR may initially inhibit mesenchymal cell differentiation since OCN-immunolabelled cells in NeoFlo CTR capsules were only seen at 60 days. The addition of 0.12% CHX gluconate, an antimicrobial agent, to the ProRoot® MTA induced apoptosis of macrophages and fibroblasts, indicating that this antimicrobial agent may cause a delay in the tissue repair and, consequently, may interfere with the bioactive potential of the materials.^20^

In contrast, birefringent and von Kossa-positive structures were observed in the capsules around all sealers at all time intervals. Considering that the von Kossa reaction is a histochemical method that detects deposits of calcium^21^, these findings suggested that these sealers can promote the precipitation of calcium in the connective tissues. The formation of calcium carbonate either as calcite or aragonite was demonstrated on the surface of NeoFlo sealer after immersion in Hanks balanced salt solution for 28 days and analyzed under micro-Raman spectroscopy^7^. A few von Kossa-positive areas were also demonstrated in the culture of BROOT-grown A4 cells, which were associated with calcium deposits probably derived from leakage and diffusion of repair material.^22,23^ Therefore, our results indicated that pure sealers or those associated with CTR allowed the deposition of amorphous calcite in the connective tissue.

The composition of endodontic sealers plays an important role in their biocompatibility and bioactive potential. Cetrimide directly influenced the inflammatory reaction of the materials, leading to greater recruitment of inflammatory cells. Despite tissue damage, all sealers evaluated induced a response in connective tissue suggestive of bioactive potential.

## Conclusion

Addition of cetrimide promoted a greater inflammatory reaction, but the reduction of inflammatory cells and rearrangement of connective tissue suggests that NeoSealer Flo, BioRoot and their associations with 1% CTR are biocompatible. Amorphous calcite deposits and OCN immunoexpression suggest that these bioceramic sealers, pure and containing 1% CTR, have bioactive potential.
